# Solution structure ensemble of human obesity-associated protein FTO reveals druggable surface pockets at the interface between the N- and C-terminal domain

**DOI:** 10.1016/j.jbc.2022.101907

**Published:** 2022-04-06

**Authors:** Balabhadra Khatiwada, Trang T. Nguyen, Jeffrey A. Purslow, Vincenzo Venditti

**Affiliations:** 1 Department of Chemistry, Iowa State University, Ames, Iowa, USA; 2 Roy J. Carver Department of Biochemistry, Biophysics and Molecular Biology, Iowa State University, Ames, Iowa, USA

**Keywords:** NMR, accelerated MD, RNA demethylase, invisible state, protein folding, protein dynamic, drug screening, αKG, α-ketoglutarate, aMD, accelerated molecular dynamics, ARTSY, amide RDC by TROSY spectroscopy, cFTO, C-terminal FTO, CPMG, Carr–Purcell–Meinboom–Gill, m6A, N6-methyladenosine, m6Am, N6, 2-O-dimethyladenosine, MD, molecular dynamics, nFTO, N-terminal FTO, PDB, Protein Data Bank, RDC, residual dipolar coupling, TROSY, transverse relaxation optimized spectroscopy

## Abstract

The fat mass and obesity-associated FTO protein catalyzes demethylation of the N^6^-methyladenosine, an epigenetic mark that controls several metabolic pathways by modulating the transcription, translation, and cellular localization of RNA molecules. Since the discovery that its overexpression links to the development of obesity and cancer, FTO was the target of screening campaigns and structure-based drug design efforts. Although several FTO inhibitors were generated, these often lack potency or selectivity. Herein, we investigate the structure and dynamics of human FTO in solution. We show that the structure of the catalytic N-terminal domain is unstable in the absence of the C-terminal domain, which explains why the isolated N-terminal domain is incompetent for catalysis and suggests that the domain interaction represents a target for the development of specific inhibitors. Then, by using NMR relaxation measurements, we show that the interface between the FTO structural domains, the active site, and several peripheral loops undergo conformational dynamics on both the picosecond–nanosecond and microsecond–millisecond timescales. Consistent with this, we found that the backbone amide residual dipolar couplings measured for FTO in phage *pf1* are inconsistent with the static crystal structure of the enzyme. Finally, we generated a conformational ensemble for apo FTO that satisfies the solution NMR data by combining the experimental residual dipolar couplings with accelerated molecular dynamics simulations. Altogether, the structural ensemble reported in this work provides an atomic-resolution model of apo FTO and reveals transient surface pockets at the domain interface that represent potential targets for the design of allosteric inhibitors.

FTO is a member of the Alkb family of nonheme Fe(II)- and α-ketoglutarate (αKG)-dependent dioxygenases and catalyzes oxidative demethylation of single-stranded RNAs *via* two coupled reactions, referred to as the primary and secondary reaction, respectively (1, 2, 3). In the secondary reaction, the αKG (secondary substrate) is reduced to succinic acid and carbon dioxide, while the metal center is oxidized to form an Fe(IV)=O species. In the primary reaction, the oxyferryl species oxidizes the methylated base (primary substrate) to reestablish the canonical nucleic acid. Although FTO was reported to be active against several methylated nucleobases, including the 3-methyluracil (4), 3-methylthymidine (4), 1-methyladenosine (5), N^6^, 2-O-dimethyladenosine (m^6^A_m_) (5, 6), N^6^-methyldeoxyadenosine (7), and the N^6^-methyladenosine (m^6^A), recent evidences suggest the m^6^A and cap m^6^A_m_ in mRNA, m^6^A and m^6^A_m_ in small nuclear RNA, and 1-methyladenosine in tRNA as the physiological substrates of the enzyme (5, 6, 7). Consequently, FTO is investigated to understand the molecular mechanisms regulating gene expression and the cellular localization of RNA molecules. In addition, FTO has attracted considerable attention as a pharmaceutical target because of the discovery that its overexpression links to the development of metabolic diseases such as obesity and cancers (8, 9, 10, 11, 12, 13).

The atomic-resolution structure of FTO has been deeply investigated by X-ray crystallography, and several crystal structures of FTO in complex with a variety of substrate analogs and inhibitors are available in the Protein Data Bank (PDB) (7, 14, 15, 16, 17, 18, 19, 20, 21). Analysis of these structures reveals that FTO is comprised of two structural domains separated by an unstructured eight-residue linker. The N-terminal domain (residues 1–322) is competent for catalysis and contains the binding site for the metal cofactor, αKG, and the methylated nucleobase. The C-terminal domain (residues 331–505) does not contact the primary or secondary substrate of FTO but forms an extensive interaction with the N-terminal domain. While the C-terminal domain is required for the correct functioning of FTO (14), its exact role in catalysis is still unknown.

Here, we investigate the apo form of human FTO and of the isolated N-terminal FTO (nFTO) and C-terminal FTO (cFTO) domains of the enzyme by solution NMR and molecular dynamics (MD) simulations. We show that the interaction between the N- and C-terminal domain is essential to stabilize the structure of the catalytic domain in its active conformation. In addition, by using NMRrelaxation experiments, we establish that FTO is a highly flexible enzyme displaying conformational dynamics both on the picosecond–nanosecond and on the microsecond–millisecond timescale. We then obtained an ensemble representation of FTO conformations in solution by combining residual dipolar couplings (RDCs) with accelerated molecular dynamics (aMD) simulations. Our data indicate that the interface between the FTO domains is more disordered than what observed in the crystal state and undergoes structural fluctuations that result in formation of large surface pockets that can accommodate small-molecule ligands. As the interaction between the N- and C-terminal domain of FTO is crucial for catalysis, these transient pockets can provide the binding site for allosteric inhibitors of the enzyme. This study highlights the ability of solution NMR and MD simulations to characterize structural disorder in proteins and to identify low-population states that are invisible to crystallography and open new possibilities for drug discovery.

## Results

In this study, we investigated a construct of human FTO in which the first 31 residues are truncated. This construct was shown to retain full enzymatic activity (14) and was employed in all crystallographic investigations of FTO. The truncated FTO will be referred to as full-length FTO (as opposed to the isolated nFTO and cFTO domains) in the rest of the article.

### The interdomain interaction is required to stabilize the structure of the catalytic domain

**Figure 1:**
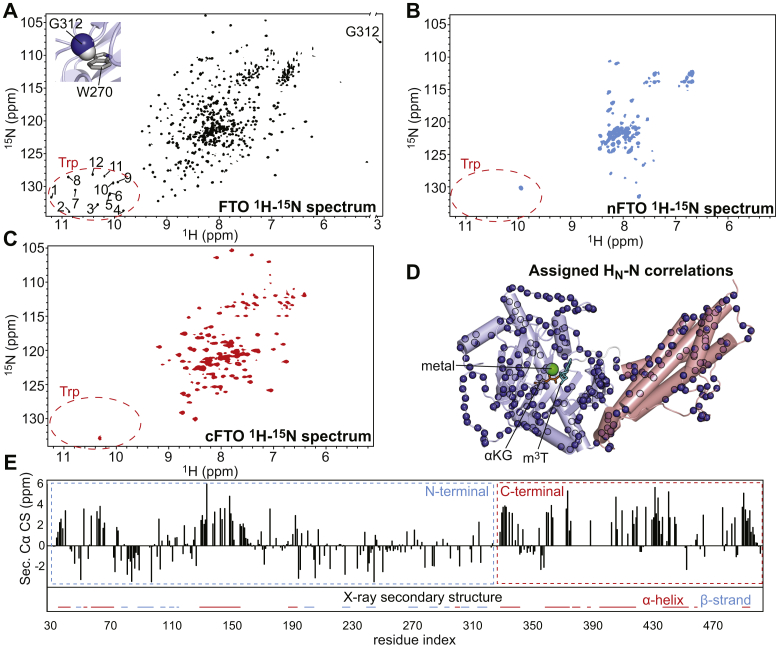
**Solution NMR of apo FTO.** TROSY spectra (800 MHz ^1^H–^15^N) of (*A*) FTO, (*B*) nFTO, and (*C*) cFTO. The spectral region containing the Nε1–Hε1 correlation from the Trp side chains is circled using a *red dashed line*. A close-up view of the interaction between the amide group of Gly^312^ and the side chain of Trp^270^ is shown in the *top left corner* of the FTO spectrum. *D*, the nitrogen atoms of the backbone amide groups unambiguously assigned in the ^1^H–^15^N TROSY spectrum of FTO are shown as *blue spheres* on the crystal structure of FTO bound to Fe(II) (*green sphere*), an αKG analog (N-oxalylglycine, *orange sticks*), and m^3^T (*cyan sticks*). The coordinates of the flexible loops missing from the crystal structure were calculated using the I-TASSER server. The N- and C-terminal domains are colored *light blue* and *salmon*, respectively. *E*, secondary Cα chemical shifts *versus* residue index. The secondary structure of FTO calculated from the X-ray coordinates is reported for comparison. Residues adopting α-helix (residues 37–45, 54–56, 59–74, 85–87, 131–158,190–196, 301–304, 331–344, 361–377, 379–384, 389–391, 397–421, 439–456, 460–462, and 492–497) or β-strand (residues 49–52, 79–83, 90–100, 105–108, 111–117, 201–207, 226–231, 270–276, 284–289, 294–297, 305–310, and 316–322) geometries are indicated as *red* and *light blue lines*, respectively. cFTO, C-terminal FTO; αKG, α-ketoglutarate; m^3^T, 3-methylthymidine; nFTO, N-terminal FTO; TROSY, transverse relaxation optimized spectroscopy.

The 800 MHz ^1^H–^15^N transverse relaxation optimized spectroscopy (TROSY) spectrum (22) of ^2^H,^15^N-labeled FTO is shown in Figure 1*A*. Preliminary analysis of the NMR data reveals the presence of 12 signals in the region occupied by the Nε1–Hε1 correlation from the Trp side chain. Since the spectrum is well dispersed and the primary sequence of FTO contains exactly 12 Trp residues, these data suggest that the enzyme is well folded in solution. Of note, we observe the presence of one NH correlation with ^1^H chemical shift of 2.9 ppm. Assignment of the NMR peaks (see later) reveals that this correlation belongs to Gly^312^ (Fig. S1). Interestingly, the amide hydrogen of Gly^312^ is packed against the side chain of Trp^270^ in the crystal structure of FTO (Fig. 1*A*, *upper left corner*). Since the ring current from aromatic groups can result in substantial shift of the NMR resonances, the observation of a large upfield shift for the ^1^H chemical shift of Gly^312^ is consistent with the crystal structure of the enzyme.

The ^1^H–^15^N TROSY spectra acquired for the ^2^H,^15^N-labeled nFTO (Fig. 1*B*) and cFTO (Fig. 1*C*) domains are of much lower quality compared with the spectrum measured for the full-length enzyme (Fig. 1*A*). In particular, the NMR peaks are considerably broader and less disperse in the isolated domains than in the full-length protein. In addition, only one Nε1–Hε1 correlation is observed in the Trp side-chain region of the nFTO and cFTO spectra. These data indicate that the isolated nFTO and cFTO are structurally unstable and that the extensive interaction between the N- and C-terminal domain of FTO is absolutely required to fold the enzyme in its functional conformation.

Assignment of the ^1^H_N_, ^15^N_H_, ^13^C_α_, ^13^C_β_, and ^13^C′ resonances of FTO was performed using triple resonance methods (23) with TROSY readout. Selective ^15^N-labeling of nine amino acids (Arg, Asn, His, Ile, Lys, Leu, Phe, Tyr, and Val) was used to resolve ambiguous assignments (Fig. S2) (24). About 426 of 449 expected peaks were observed in the ^1^H–^15^N TROSY spectrum of FTO (note that the 25 Pro residues are not expected to provide a backbone amide peak). A total of 248 NH correlations were unambiguously assigned (∼55% of the expected peaks). The low assignment rate is due to the large size of the enzyme (54 kDa), its unfavorable relaxation properties, and the inability to produce stable samples of FTO at high concentrations (note that the assignment experiments were measured on samples containing ∼0.3 mM FTO). Nonetheless, the assigned resonances are homogenously distributed on the enzyme structure and cover numerous areas of interest (Figs. 1*D* and S3). In particular, several assigned amide correlations are localized at the interface between the N- and C-terminal domain of the enzyme, within and surrounding the binding site for the primary and secondary substrates, within the nucleotide recognition loop (residues 213–225) and within unstructured loops that are not observed by crystallography because of the lack of electron density (residues 121–129, 159–188, and 251–263). Of note, the distribution of secondary C_α_ chemical shifts along the FTO primary sequence is consistent with the secondary structure calculated from the crystal structure of the holo enzyme (Fig. 1*E*), which provides further evidence that the overall fold of apo FTO in solution resembles the one observed for the holo enzyme in the crystal state. The assigned backbone resonances for FTO were deposited on the BioMagResBank (accession number: 51176) (25).

### Solution structure ensemble of apo FTO indicates a flexible interdomain interface

To better investigate the consistency of the crystal structure of holo FTO with the solution structure of the apo enzyme, we have measured backbone amide ^1^D_NH_ RDC data for apo FTO partially aligned in a dilute liquid crystalline medium of phage *pf1* (26). ^1^D_NH_ RDCs provide information on the orientation of the N–H bond vectors relative to the external magnetic field and are commonly employed to assess the quality of and refine crystallographic structures (27).

**Figure 2:**
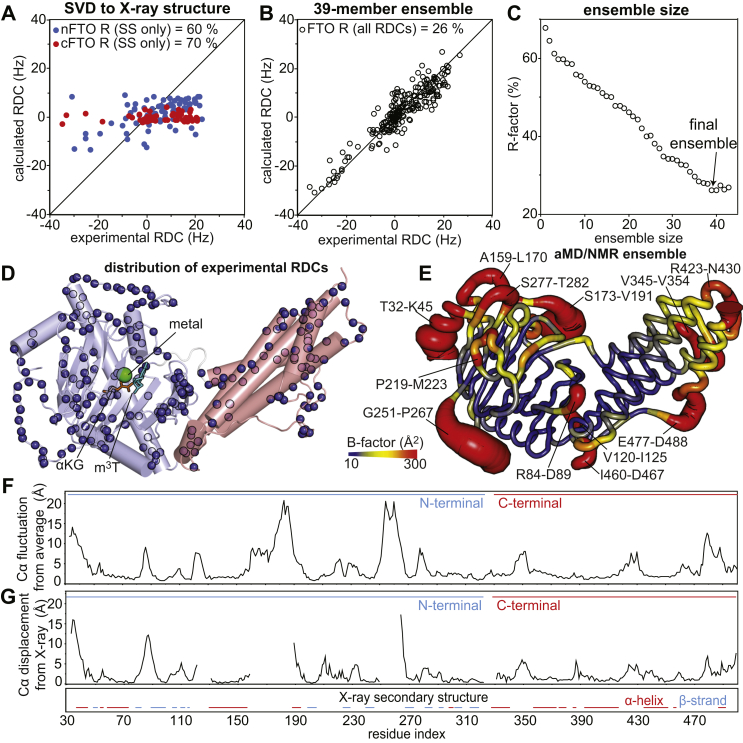
**Solution conformational ensemble for apo FTO.***A*, comparison of the observed and calculated RDCs obtained by SVD of the data coming from secondary structures of the N- (80 RDC values, *blue circles*) or C-terminal (55 RDC values, *red circles*) domain to the coordinates of the FTO crystal structure (Protein Data Bank code: 3LFM). *B*, agreement between the full set of 226 experimental RDCs (including secondary structures and unstructured loops in both FTO domains) and the RDCs back-calculated from the 39-memeber aMD/NMR ensemble. The error on the experimental RDCs was computed from the signal-to-noise ratio of the ARTSY spectra (28). The error was <2 Hz for every analyzed RDC value. *C*, *R*-factor *versus* ensemble size for the aMD/NMR ensemble refinement of apo FTO. A 39-member ensemble is required to fulfil the experimental RDCs. *D*, the backbone amides for which RDC data were obtained and used in the ensemble calculation are shown as *blue spheres* on the crystal structure of FTO bound to Fe(II) (*green sphere*), an αKG analog (N-oxalylglycine, *orange sticks*), and m^3^T (*cyan sticks*). The coordinate of the flexible loops missing from the crystal structure were calculated using the I-TASSER server. The N- and C-terminal domains are colored *light blue* and *salmon*, respectively. *E*, sausage representation of the aMD/NMR ensemble. Cartons are colored according to *B*-factor, as indicated by the color bar. *B*-factors were calculated using the formula $B_{i}=8{\text{π}}^{2}U_{i}^{2}$, where *B*_*i*_ and *U*_*i*_ are the *B*-factor and mean-square displacement of atom *i*, respectively. *F*, plot of the Cα atomic fluctuation from the average structure in the ensemble *versus* residue index. *G*, the Cα displacement from the X-ray structure calculated for the representative N- and C-terminal domain structure of the conformational ensemble is plotted *versus* the residue index. The secondary structure of FTO calculated from the X-ray coordinates is reported for comparison. Residues adopting α-helix (residues 37–45, 54–56, 59–74, 85–87, 131–158,190–196, 301–304, 331–344, 361–377, 379–384, 389–391, 397–421, 439–456, 460–462, and 492–497) or β-strand (residues 49–52, 79–83, 90–100, 105–108, 111–117, 201–207, 226–231, 270–276, 284–289, 294–297, 305–310, and 316–322) geometries are indicated as *red* and *light blue lines*, respectively. aMD, accelerated molecular dynamics; ARTSY, amide RDC by TROSY spectroscopy; αKG, α-ketoglutarate; m^3^T, 3-methylthymidine; RDC, residual dipolar coupling; SVD, singular value decomposition.

We have measured ^1^D_NH_ RDC data for 144 and 79 nonoverlapping NMR signals coming from the N- and C-terminal domain of full-length FTO (plus three RDCs coming from the flexible linker), respectively, by using the amide RDCs by TROSY spectroscopy (ARTSY) pulse sequence (Fig. 2*D*) (28). Interestingly, singular value decomposition fitting of the data coming from secondary structures (80 and 55 RDC values for the N- and C-terminal domain, respectively) to the coordinates of the N- and C-terminal domains of the holo FTO X-ray structure returns *R*-factors of 60 and 70%, respectively (Fig. 2*A*). The poor agreement between experimental and back-calculated data indicates that no single orientation of the atomic coordinates in the PDB file of holo FTO (PDB code: 3LFM) can be found that satisfies the experimental RDC data, and, therefore, the crystal structure of holo FTO does not fully capture the behavior of the apo enzyme in solution. Since Alkb enzymes are known to be highly flexible proteins (29, 30, 31, 32), we ascribe the inconsistency between the crystal structure and solution NMR data to conformational dynamics.

To obtain a structural model of apo FTO in solution that would account for conformational dynamics, we have calculated a structural ensemble for the enzyme by coupling the experimental ^1^D_NH_ RDCs with aMD simulations (31). We have proven this protocol successful in generating MD-derived structural ensembles of dynamical proteins that satisfy solution NMR data (31, 32, 33). An ensemble of 39 conformations extracted from the aMD trajectory is required to fulfill the entire set of 226 experimental RDCs (including the data from unstructured regions) (Fig. 2, *B* and *C*). The obtained structure ensemble confirms that FTO is a highly flexible enzyme (Fig. 2, *E* and *F*). Indeed, regions with high conformational disorder (resulting in large *B*-factor) are observed at the N terminus (residues 32–45), peripheral loops (residues 173–191, 251–267, 277–282, 345–354, 423–430, and 477–488), and loops located at the interface between the N- and C-terminal domain (residues 84–89, 120–125, and 460–467) (Fig. 2, *E* and *F*). It is also important to highlight that an overlay of the crystal structure of holo FTO with the representative structure of the conformational ensemble calculated for apo FTO (*i.e.*, the ensemble member with the lowest backbone rmsd from the average structure calculated over the ensemble) reveals large C⍺ rmsd at several flexible loops located on both the N- and C-terminal domain of the enzyme (Figs. 2*G* and S4). Although these results may underline ligand-induced conformational changes, it should be noted that these discrepancies between crystal structure and solution structure ensemble could be artifacts deriving from crystal packing.

### NMR relaxation shows that FTO is dynamic on the picosecond–nanosecond and microsecond–millisecond timescale

NMR relaxation experiments are a preferred tool for experimental investigations of protein conformational dynamics. In particular, measuring the longitudinal (*R*_*1*_) and transverse (*R*_*2*_) relaxation rates reports on the regions of the protein that are flexible on the picosecond–nanosecond timescale (34). Relaxation dispersion experiments inform on areas of the protein structure that undergo conformational dynamics on the microsecond–millisecond timescale (34, 35).

**Figure 3:**
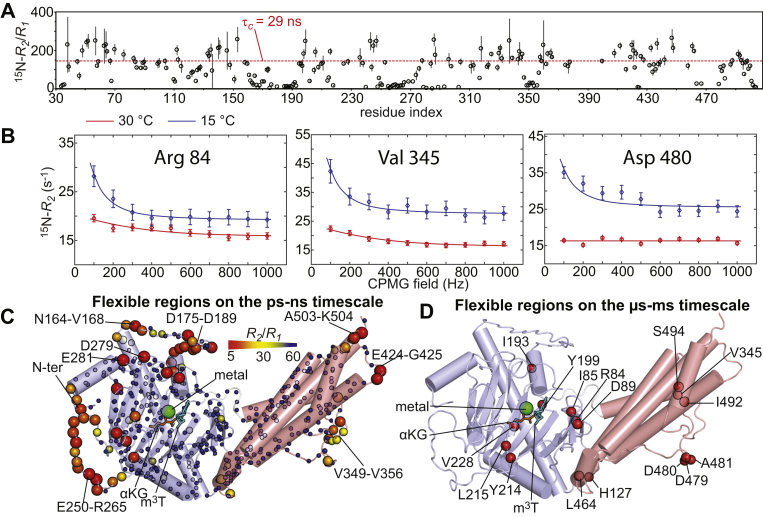
**FTO is dynamic on picosecond–nanosecond and microsecond–millisecond timescale.***A*, ^15^N *R*_*2*_/*R*_*1*_ ratios measured for apo FTO at 800 MHz and 30 °C are plotted *versus* residue index. The *dashed red line* indicates the expected *R*_*2*_/*R*_*1*_ ratio for a rigid globular protein the size of FTO. *B*, examples of typical 800 MHz relaxation dispersion data measured at 15 (*blue*) and 30 °C (*red*). Data are shown for Arg^84^, Val^345^, and Asp^480^ with the experimental data represented as *circles* and the best-fit curves as *solid lines*. *Curves* for all residues displaying significant relaxation dispersion are shown in Fig S5. *C*, the *R*_*2*_/*R*_*1*_ values measured for the apo enzyme are plotted on structure of FTO. The relationship between experimental values and the color and size of the spheres is provided by the color bar. *D*, backbone amides displaying significant ^15^N relaxation dispersion at 15 °C are shown as *red spheres* on the structure of FTO. In *C* and *D*, the crystal structure of FTO bound to Fe(II) (*green sphere*), an αKG analog (N-oxalylglycine, *orange sticks*), and m^3^T (*cyan sticks*) is shown. The coordinate of the flexible loops missing from the crystal structure were calculated using the I-TASSER server. The N- and C-terminal domains are colored *light blue* and *salmon*, respectively. αKG, α-ketoglutarate; m^3^T, 3-methylthymidine.

Residue-specific ^15^N *R*_*1*_ and *R*_*2*_ values were measured at 800 MHz and 30 °C by acquisition of TROSY-detected *R*_*1*_ and *R*_*1ρ*_ experiments (36) on ^2^H, ^15^N-labeled FTO and are reported as ^15^N-*R*_*2*_/*R*_*1*_ ratios in Figure 3, *A* and *C*. For a rigid protein, where global rotational tumbling is the only contribution to the picosecond–nanosecond dynamics, the *R*_*2*_/*R*_*1*_ values are expected to be constant throughout the primary sequence and proportional to the rotational correlation time (*τ*_*c*_) (37). Instead, the presence of flexible structural elements within the protein (such as long and flexible loops) that locally increase the picosecond–nanosecond dynamics experienced by the backbone amide groups is revealed by a local shift of the *R*_*2*_/*R*_*1*_ ratios toward lower than average values (37). At 30 °C, a globular protein of the size of FTO (54 kDa) is expected to have *τ*_*c*_ ∼ 29 ns (see the Experimental procedures section), which translates to an 800 MHz ^15^N-*R*_*2*_/*R*_*1*_ ratio of ∼147. The average ^15^N-*R*_*2*_/*R*_*1*_ ratio measured for FTO is 107 ± 75, which is consistent with the predicted *τ*_*c*_ value (Fig. 3*A*). Interestingly, analysis of the ^15^N-*R*_*2*_/*R*_*1*_ values *versus* residue index (Fig. 3, *A* and *C*) reveals the presence of several residues with a lower than average ^15^N-*R*_*2*_/*R*_*1*_. These residues are localized at the N- and C-terminal ends of the protein (residues 34–36 and 502–504, respectively) and within the peripheral loops displaying large *B*-factors in the RDC/aMD conformational ensembles (residues 164–193, 248–265, 279–285, 349–356, and 424–425) (compare Figs. 2*E* and 3*C*).

Relaxation dispersion data (800 MHz ^15^N) were measured on ^2^H, ^15^N-labeled FTO at 30 and 15 °C using the Carr–Purcell–Meinboom–Gill (CPMG) experiment (35, 38). Significant relaxation dispersion was observed for the backbone amides of 16 residues (Figs. 3, *B* and *D* and S5). Of note, six of these residues (I193, Y199, Y214, L215, V228, and V345) do not fall in a well-defined area of FTO but are scattered within the protein structure (Fig. 3*D*). We ascribe the relaxation dispersions observed at these residues to local microsecond–millisecond timescale structural fluctuations that affect the ^15^N chemical shift of a single amide group (*i.e.*, formation/disruption of a hydrogen bond and/or rearrangement of a nearby side chain). On the other hand, the remaining 10 relaxation dispersions cluster at the interface between the N- and C-terminal domain (R84, I85, D89, H127, and L464) and at the loop connecting α-helix 11 to α-helix 12 on the C-terminal domain (D479, D480, A481, I492, and S494) (Fig. 3*D*). Consistent with the relaxation dispersion results, both these regions display a high degree of disorder in the calculated NMR/aMD FTO ensemble (Fig. 2, *E* and *F*).

Quantitative analysis of the relaxation dispersion curves using the Carver–Richards equation indicates that the data can be globally fit to a two-site exchange model in which the protein is in equilibrium between two conformational states with different ^15^N chemical shifts (Fig. S5). The best fit exchange rate (*k*_*ex*_) values are ∼500 and ∼100 s^−1^ at 30 and 15 °C, respectively. However, it should be noted that because of the fact that FTO is a large enzyme with fast ^15^N-*R*_*2*_ rates, the lowest refocusing field that we were able to access is 100 Hz (Fig. 3*B*). This experimental factor limits the accuracy of the quantitative modeling of the relaxation dispersion experiments.

## Discussion

Pharmacological inhibition of the fat mass and obesity-associated FTO protein is emerging as a promising strategy to develop a therapeutic treatment for obesity and cancer (8, 9, 10, 11, 12, 13), and several inhibitors of the enzyme have been described in the literature (15, 20, 21, 39, 40, 41, 42, 43, 44, 45, 46). However, the majority of the reported inhibitors lack potency or FTO selectivity over other Alkb demethylases. As of today, only four selective inhibitors of FTO were identified *via* a virtual screening campaign and structure-based design (46, 47, 48).

In this work, we have investigated the solution structure and dynamics of the apo FTO by solution NMR and MD simulations. By comparing the NMR spectra acquired for the full-length enzyme with the ones measured for the isolated nFTO and cFTO, we have shown that the interaction between the FTO structural domains is absolutely required to stabilize the structure of the catalytic N-terminal domain (Fig. 1). This observation sheds light on the function of the C-terminal domain and is consistent with previous work reporting that the isolated nFTO is enzymatically inactive and that single-point mutations at the interface between the N- and C-terminal domain abolish the activity of the full-length enzyme (14). Since the FTO C-terminal domain has a unique fold and it is not present in other members of the Alkb dioxygenases family (14), our data suggest the interface between the N- and C-terminal domain as a target for developing selective FTO inhibitors.

**Figure 4:**
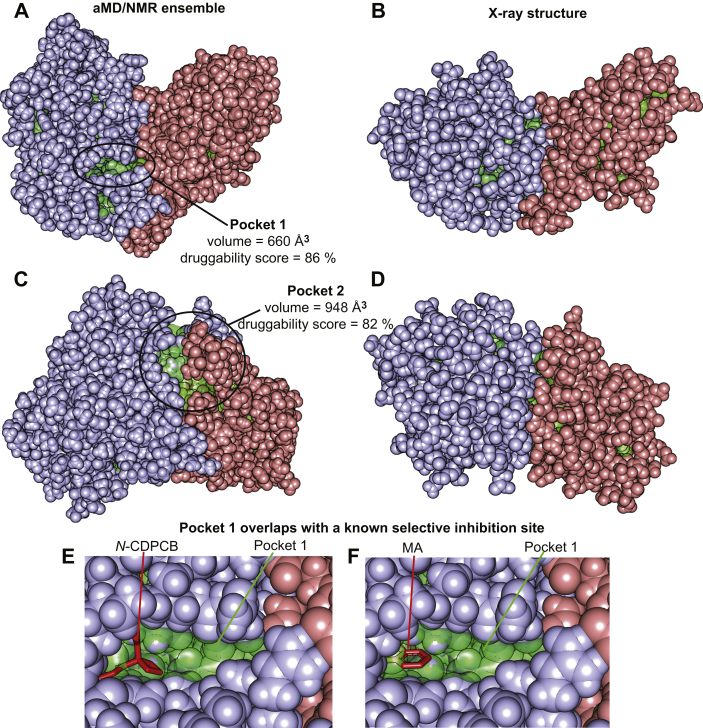
**Two druggable pockets at the interdomain interface.** Two structures extracted from the aMD/NMR ensemble (*A* and *C*) are compared with two identical orientations of the crystal structure of FTO (*B* and *D*, respectively). The N- and C-terminal domains are colored *light blue* and *salmon*, respectively. The surface pockets detected by the Pymol software are highlighted in *green*. In the structure ensemble, two large pockets are identified at the interface between the N- and C-terminal domain that are not present in the crystal structure. Volume and druggability score for the two pockets calculated by the DoGSiteScorer server are reported. In *E* and *F*, the selective FTO inhibitors *N*-CDPCB and MA, respectively, are placed in pocket 1 (the small molecules are shown as *red sticks*). The modeling was performed by superimposing the crystal structure of the FTO-inhibitor complex (Protein Data Bank code: 5DAB and 3QKN, respectively) onto the structure extracted from the conformational ensemble. aMD, accelerated molecular dynamics; MA, meclofenamic acid; *N*-CDPCB, *N*-(5-chloro-2,4-dihydroxyphenyl)-1-phenylcyclobutanecarboxamide.

Analysis of the ^15^N *R*_*1*_, *R*_*2*_, and relaxation dispersion NMR data indicated that FTO experiences conformational dynamics on both the picosecond–nanosecond and microsecond–millisecond timescale (Fig. 3). In agreement with this finding, the ^1^D_NH_ RDC data measured for FTO by solution NMR are inconsistent with the crystallographic structure of the enzyme (Fig. 2*A*). With the help of all-atom aMD simulations, we generated a 39-member conformational ensemble of apo FTO that satisfies the experimental ^1^D_NH_ RDCs (Fig. 2, *B* and *C*). Examination of the conformational ensemble revealed that while the overall tertiary structure closely resembles the one seen in the crystal state, several loops are highly disordered in solution (Fig. 2, *E* and *F*). Of note, three of these flexible loops are integral part of the interface between the N- and C-terminal domain (Fig. 2*E*), indicating that conformational dynamics modulate the interaction between the FTO domains. Such conformational variability of the domain–domain interface of FTO was not apparent from the crystallographic studies reported so far on the enzyme (Fig. S6) and suggests that FTO functional regulation can be achieved by allosteric perturbations of protein dynamics. Interestingly, analysis of the conformational ensemble highlights formation of two large surface pockets that are deemed druggable by the DoGSiteScorer prediction server (druggability score of 86 and 82%, respectively) (Fig. 4) (49). Since the pockets identified here are located at the interface between the N- and C-terminal domain and are invisible in the crystal structure (Fig. 4), our study provides the basis for virtual screening efforts aimed at discovering a new class of allosteric inhibitors of FTO that disrupt the enzymatic activity by perturbing the interdomain interaction. Of note, pocket 1 revealed by our study (Fig. 4) overlaps with the biding site for the selective FTO inhibitors *N*-(5-chloro-2,4-dihydroxyphenyl)-1-phenylcyclobutanecarboxamide (40) and meclofenamic acid (21) (Fig. 4, *E* and *F*), indicating that the observed transient pockets can provide the interaction site for novel allosteric inhibitors. It is also important to stress out that the binding site for *N*-(5-chloro-2,4-dihydroxyphenyl)-1-phenylcyclobutanecarboxamide and meclofenamic acid is not observed in any of the structures of FTO crystallized in the absence of these inhibitors, further supporting the hypothesis that the available crystal structures of FTO do not provide a comprehensive picture of the available small-molecule binding sites on the enzyme.

In conclusion, our work revealed that the function of the FTO C-terminal domain is to stabilize the structure of the catalytic N-terminal domain, identified surface pockets at the domain–domain interface that can be the target for allosteric inhibitors of FTO, and highlighted the utility of combining NMR and MD data to detect and visualize conformational disorder in proteins.

## Experimental procedures

### Expression and purification

The FTO construct used in this study (residues 32–505, see Results section for justification) was expressed and purified as described previously (50). In brief, a plasmid containing the sequences of FTO, an N-terminal His tag, an N-terminal EIN-fusion solubility tag, and the tobacco etch virus (TEV) protease consensus sequence (His_6_-EIN-TEV-FTO) was transformed into the BL21 star (DE3) *Escherichia coli* cells. A single colony from the plated cells was grown in a M9 minimal medium at 37 °C and 180 rpm. Uniform ^2^H, ^15^N isotopic labeling was obtained by using 99.9% D_2_O as the solvent and ^2^H glucose and ^15^NH_4_Cl as sole carbon and nitrogen sources, respectively. Uniform ^2^H, ^13^C, ^15^N isotopic labeling was obtained by using 99.9% D_2_O as the solvent and ^2^H, ^13^C glucose, and ^15^NH_4_Cl as sole carbon and nitrogen sources, respectively. Selective ^15^N labeling of Arg, Asn, Gln, His, Lys, Phe, Tyr, and Trp residues was achieved by introducing 100 mg/l of the ^15^N labeled amino acid of interest to the M9 medium 1 h before induction. Selective ^15^N labeling of Ile, Leu, and Val residues was performed within the same NMR sample by adding 100 mg/l of each ^15^N labeled amino acid to the same M9 medium 1 h before induction (Figs. S2 and S7). At absorbance of ∼0.6 at 600 nm, the expression was induced with 1 mM IPTG, and the cells were incubated at 16 °C and 180 rpm for 16 h. Then, the cells were harvested, suspended in 20 ml of 50 mM Tris–HCl (pH 8.0) and 500 mM NaCl, and lysed using an EmulsiFlex-C3 microfluidizer (Avestin). The lysate was centrifuged at 20,000*g* for 30 min, and the supernatant was filtered using 0.45 μm filter before loading on a HisTrap HP column (5 ml; GE Healthcare). The protein was eluted with a 100 ml gradient of 375 mM imidazole and 500 mM NaCl in Tris–HCl (pH 8.0). The EIN fusion tag was removed by digestion with the 0.25 mg TEV protease at room temperature for ∼6 h and repassing through the HisTrap HP column. The protein was further purified by using a Superdex75 gel filtration column (GE Healthcare) equilibrated with 20 mM Tris–HCl (pH 7.4), 200 mM NaCl, 2 mM DTT, and 1 mM EDTA. Finally, the sample was passed through an ENrich Q ion exchange column using a 0 to 100 ml gradient of 20 mM Tris–HCl (pH 7.4), 1 M NaCl, 2 mM DTT, and 1 mM EDTA.

The isolated nFTO (residues 32–326) and cFTO (residues 327–505) were expressed with the His_6_-EIN-fusion plasmid and purified using the same protocol as the full-length enzyme, with the exception that TEV cleavage was performed at 4 °C and pH 8.0 for 13 h in 20 mM Tris–HCl (pH 8.0) and 50 mM NaCl.

### NMR experiments

NMR samples containing ∼0.3 mM FTO were prepared in 20 mM Tris–HCl (pH 7.4), 100 mM NaCl, 0.02% NaN_3_, 1 mM EDTA, 2 mM DTT, 1× EDTA-free protease inhibitor, and 90% H_2_O/10% D_2_O (v/v). Samples containing cFTO were prepared at pH 8.0.

All NMR spectra were acquired at 30 °C on Bruker 600, 700, and 800 MHz spectrometers equipped with Z-shielded gradient triple resonance cryoprobes. Spectra were processed and analyzed using the program NMRPipe (51) and SPARKY (http://www.cgl.ucsf.edu/home/sparky), respectively.

Backbone resonance assignment was performed using TROSY versions of conventional 3D triple resonance correlation experiments (HNCO, HNCA, HN(CO)CA, HNCACB, and HN(CO)CACB) (23).

Backbone amide ^1^D_NH_ RDCs were measured by taking the difference in ^1^J_NH_ scalar couplings in aligned and isotropic media. The alignment media employed were phage *pf1* (8 mg/ml; ASLA Biotech), and ^1^J_NH_ couplings were measured using the ARTSY pulse scheme (28). Singular value decomposition analysis of RDCs was carried out using XPLOR-NIH (National Institutes of Health) (52).

Backbone amide ^15^N *R*_*1*_ and *R*_*1ρ*_ experiments were carried out at 30 °C and 800 MHz using heat-compensated pulse schemes with a TROSY readout (36). The strength of the spin-lock field for the *R*_*1ρ*_ experiment was set to 1 kHz. The relaxation decay was sampled for eight delay durations of 0, 80, 200, 320, 440, 560, 720, and 840 ms for *R*_*1*_ and 0.2, 1.4, 2.4, 5.0, 7.8, 10.8, 14.0, 17.4, and 20 ms for *R*_*1ρ*_. *R*_*1*_ and *R*_*1ρ*_ values were determined by fitting the time-dependent exponential restoration of peak intensities at increasing relaxation delays. *R*_*2*_ values were extracted from the measured *R*_*1*_ and *R*_*1ρ*_ values. The ^15^N *R*_*2*_/*R*_*1*_ for a globular protein of the size of FTO (54 kDa) at 30 °C was estimated using the following equation:

(1) $$\frac{R_{2}}{R_{1}}\approx \;\frac{{\left(4\pi \nu_{N}\tau_{c}\right)}^{2}+7}{6}$$

where $\nu_{N}$ is the ^15^N resonance frequency in Hertz, and $\tau_{c}$ is the estimated rotational correlation time at 30 °C calculated as:

(2) $$\tau_{c}\left(25\;{}^{\circ}C\right)\approx 0.0005998\text{ MW}+0.1674$$

(3) $$\tau_{c}\left(30\;{}^{\circ}C\right)\approx \tau_{c}\left(25\;{}^{\circ}C\right)\frac{298\;K}{303\;K}\frac{0.7973\;cP}{0.8903\;cP}$$

where, $\tau_{c}\left(25\;{}^{\circ}C\right)$ and $\tau_{c}\left(30\;{}^{\circ}C\right)$ are the rotational correlation times at 25 and 30 °C, respectively, MW is the protein molecular weight (in Dalton), and the water viscosities at 25 and 30 °C (0.8903 and 0.7973 cP, respectively) are calculated as described by Cho *et al*. (53). Equation 2 is derived by linear fitting of the data reported in the NESG website (www.nmr2.buffalo.edu/nesg.wiki/NMR_determined_Rotational_correlation_time).

Backbone amide ^15^N CPMG relaxation dispersion experiments were carried out at 800 MHz and two temperatures (15 and 30 °C) using a pulse sequence that measures the exchange contribution for the TROSY component of the ^15^N magnetization (54). Off-resonance effects and pulse imperfections were minimized using a four-pulse phase scheme (55). Experiments were performed with a fixed relaxation delay (40 ms) but a changing number of refocusing pulses to achieve different effective CPMG fields (100, 200, 300, 400, 500, 600, 700, 800, 900, and 1000 Hz) (38). Experimental errors on the transverse relaxation rates were estimated from the noise level estimated with the SPARKY software (35). The resulting RD curves acquired at multiple temperatures were globally fit to a two-site exchange model using the Carver–Richard equation, as described previously (56, 57).

### Calculation of conformational ensembles

The conformational ensemble for apo FTO was calculated by combining aMD simulations and the NMR-derived ^1^D_NH_ RDC data, as recently described (31).

A 1 μs aMD simulation (58) was performed using the Amber 16 package (59). The X-ray structure (PDB code: 3LFM) (14) was used as a starting conformation where the residues missing from the crystal structure (122–129, 160–188, 251–263, 425–427, and 500–505) were modeled using the I-TASSER server (https://zhanggroup.org/I-TASSER/) (60). Missing hydrogen atoms were built from Leap module in AMBER16 with the FF14SB force field. The system was placed in a TIP3P water box, and the distance from the surface of the water box to all the atoms of the solute is set to 10 Å. Counterions were added to neutralize the charge. Energy minimization was carried out using the steepest descent method followed by the conjugate gradient minimization. Then the system was heated from 1 to 310 K for 1 ns and equilibrated at a constant pressure (1 atm) for 5 ns. The bonds involving hydrogen atoms were restrained by using the SHAKE algorithm. The electrostatic interactions were treated with a cutoff of 8 Å for long-range interactions using the particle-mesh Ewald summation. An integration step of 1 fs was used. The aMD simulation was run at the “dual-boost” level in which the total potential energy and the dihedral energy were boosted. A short (20 ns) MD simulation was used to collect the potential statistics for calculating aMD acceleration parameters (E_D_, α_D_, E_P_, and α_P_):

(4) $$E_{D}=E_{D^{\prime }}+\;{\text{α}}_{1}\frac{N_{res}}{5}$$

(5) $${\text{α}}_{D}=\;{\text{α}}_{1}\;\times \;\frac{N_{res}}{5}$$

(6) $$E_{P}=\;E_{P^{\prime }}+\;{\text{α}}_{2}N_{atom}$$

(7) $${\text{α}}_{P}=\;{\text{α}}_{2}\;\times \;N_{atom}$$

where *E*_*P’*_ and *E*_*D’*_ are the average potential and dihedral energy, respectively, during the 20 ns MD. *N*_*res*_ and *N*_*atom*_ are the total number of residues and atoms in the system, respectively. α_1_ (3.5 kcal mol^−1^ residue^−1^) and α_2_ (0.2 kcal mol^−1^ atom^−1^) are the approximate energy contribution per degree of freedom calculated over residues and atoms, respectively (58). The 1 μs of aMD simulation was run with *E*_*D*_ = 8034 kcal mol^−1^, E_P_ = −195,054 kcal mol^−1^, *α*_*D*_ = 379.2 kcal mol^−1^, *α*_*P*_ = 10,853 kcal mol^−1^.

To generate the structural ensemble representation, the aMD trajectory was clustered to produce representative structures with a high degree of structural diversity. Each representative structure was energy minimized, and the ensemble of representative structures was used to fit the experimental RDC data. Back-calculation of RDCs from the conformational ensembles was done using the following equation:

(8) $$RDC_{i}=\underset{k}{\sum }D_{k}\;\left[\left(3{\cos\;}^{2}\;\theta -1\right)+\;\frac{3}{2}\left(sin^{2}\theta \;\cos\;2\;\Phi \right)\right]$$

where *θ* is the angle formed between the internuclear bond vector of the amide group of residue *i* and the *z*-axis of the alignment tensor, φ is the angle between the xy plane projection of the internuclear bond vector and the *x*-axis, and *D*_*k*_ is the magnitude of the alignment tensor for ensemble member *k* multiplied by its fractional population in the ensemble. *D*_*k*_, *θ*, and φ were optimized to reduce the discrepancy between experimental and back-calculated RDCs using the MATLAB script downloadable at http://group.chem.iastate.edu/Venditti/downloads.html.

The consistency between experimental and back-calculated RDC data was evaluated in terms of *R*-factor (61):

(9) $$R-factor=\underset{i}{\sum }\sqrt{{\left(RDC_{i}^{exp}-RDC_{i}^{calc}\right)}^{2}/\left(2RD{C_{i}^{exp}}^{2}\right)}$$

where $RDC_{i}^{exp}$ and $RDC_{i}^{calc}$ are the experimental and back-calculated RDC for residue *i*, respectively. The protocol was iterated by increasing the number of clusters (and therefore the representative structures in the pool) until a stable *R*-factor was obtained.

## Data availability

All data needed to evaluate the conclusions in the article are present in the article and/or the supporting information.

## Supporting information

This article contains supporting information.

## Conflict of interest

The authors declare that they have no conflicts of interest with the contents of this article.
