# Supplementary material for: Solution structure ensemble of human obesity-associated protein FTO reveals druggable surface pockets at the interface between the N- and C-terminal domain
Source: J Biol Chem. 2022 Apr 6;298(5):101907. doi: 10.1016/j.jbc.2022.101907 (PMC9065727; doi:10.1016/j.jbc.2022.101907)
Supplement: Supplemental Figures S1–S7 [file mmc1.pdf]

SUPPLEMENTAL MATERIALS

Solution structure ensemble of human obesity-associated protein FTO reveals druggable surface pockets at the interface between the N- and C-terminal domain

Balabhadra Khatiwada[1], Trang T. Nguyen[1], Jeffrey A. Purslow[1] and Vincenzo Venditti[1,2,*]

[1] *Department of Chemistry, Iowa State University, Ames, Iowa 50011, USA.*
[2] *Roy J. Carver Department of Biochemistry, Biophysics and Molecular Biology, Iowa State University, Ames, Iowa 50011, USA.*

*Address correspondence to: Vincenzo Venditti, Department of Chemistry, Iowa State University, Hach Hall, 2438 Pammel Drive, Ames, IA 50011, USA. Email: venditti@iastate.edu; Tel: 515-294-1044; Fax: 515-294-7550; ORCID 0000-0001-8734-0400.

Running title: Solution conformational ensemble of apo FTO

Keywords: NMR, accelerated MD, RNA demethylase, invisible state, protein folding, protein dynamic, drug screening

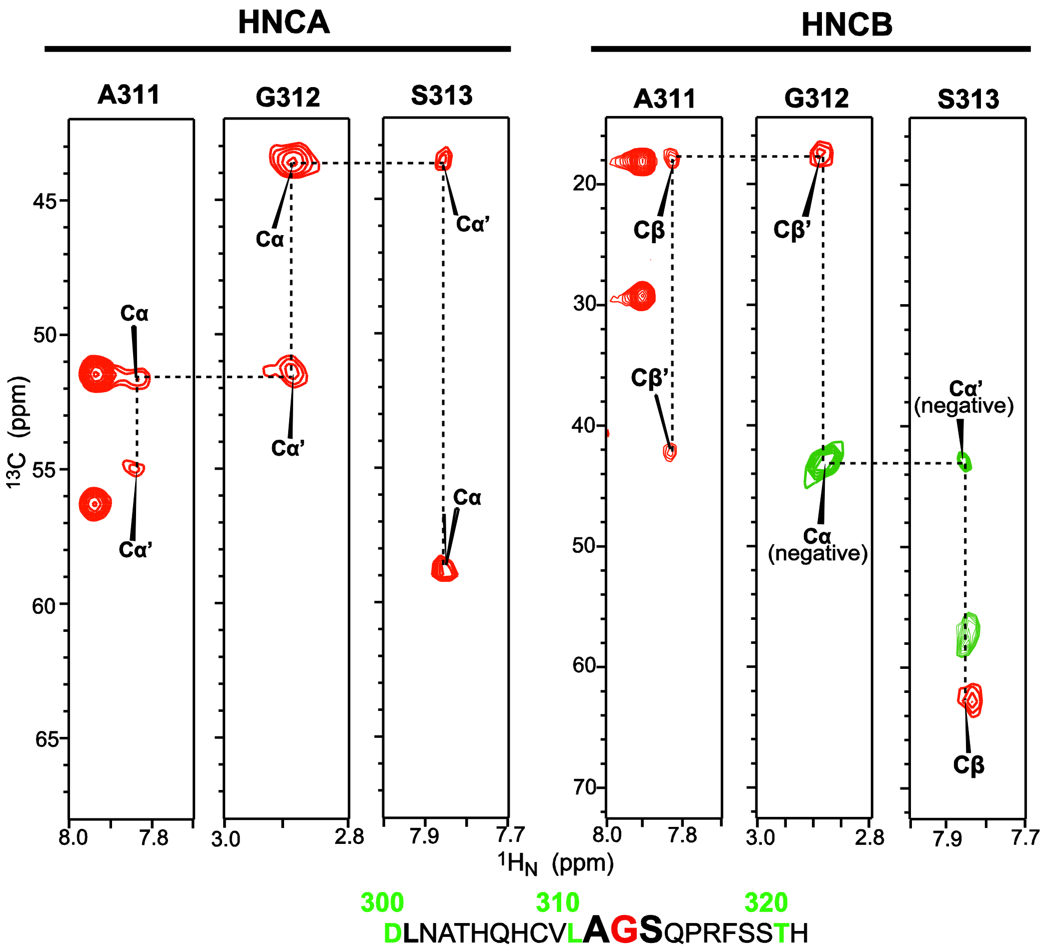

Figure S1. Assignment of the Gly[312] amide correlation. Strip plots of the HNCA (left) and HNCB (right) spectra extracted at the ^{15}N chemical shifts of the Ala[311] (left), Gly[312] (center), and Ser[313] (right) amide peaks.

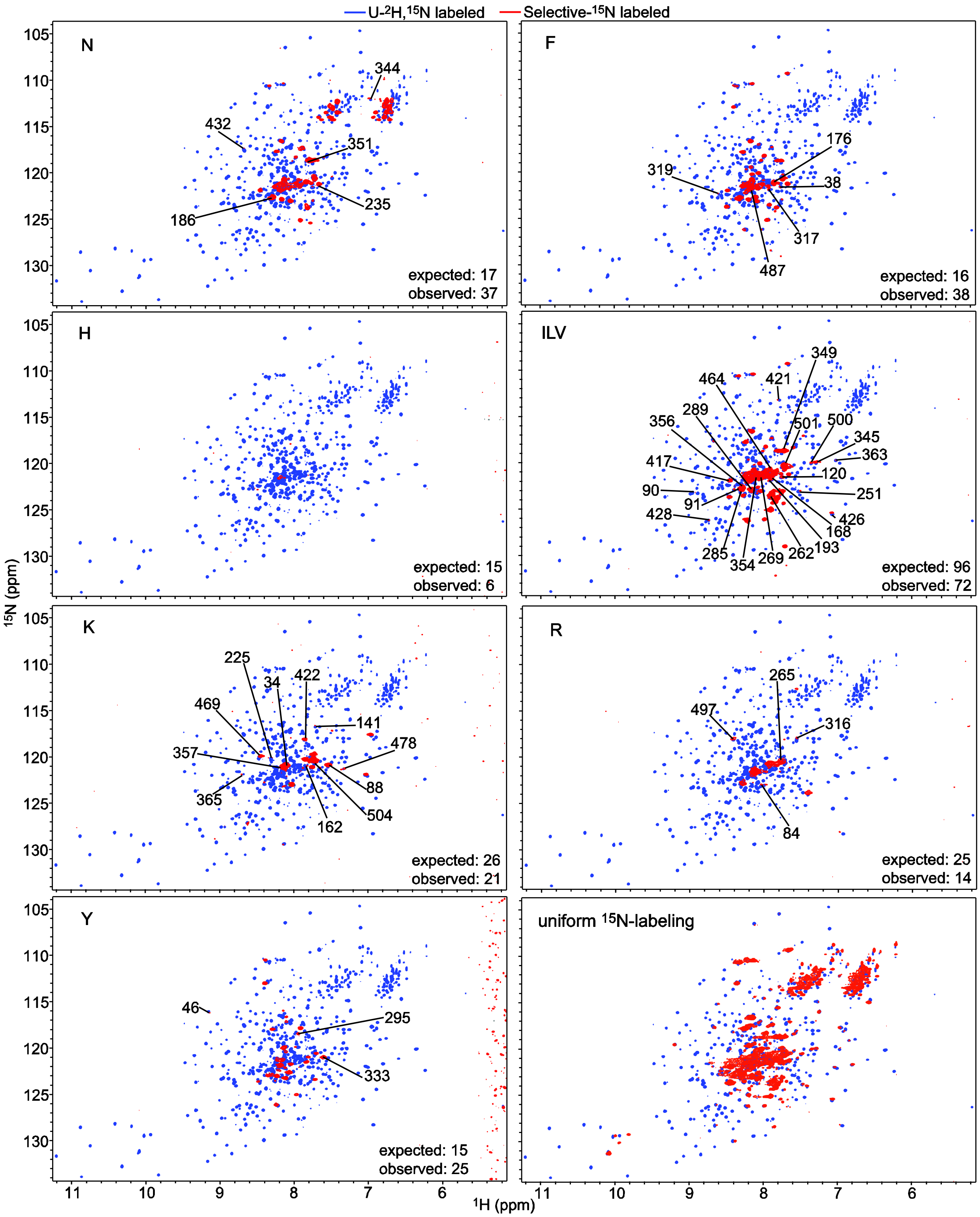

Figure S2. Selective ^{15}N-labeling of Arg, Asn, His, Ile, Lys, Leu, Phe, Tyr, and Val residues. The ^{1}H-^{15}N TROSY spectra measured for selective ^{15}N-labled FTO (red) are superimposed on the spectrum of U-^{2}H, ^{15}N-labeled FTO (blue). The selective labeled spectra are of lower quality due to the absence of deuteration in the labeling scheme (note that the ^{1}H-^{15}N TROSY spectrum of FTO is dramatically improved by deuteration – see bottom right corner). In addition, scrambling severely affects the ability to obtain unambiguous results from the selective labeling of Asn (scrambling into Ile, Leu, and Val), Phe

(scrambling into Ile, Leu, Val and Tyr), and Tyr (scrambling into Ile, Leu, Phe, and Val) residues. Therefore, the data from these selective labeling experiments were only used as ambiguous constraints to guide the sequential assignment performed using triple resonance NMR experiments. The purity and fold of the selectively labeled FTO samples were confirmed by gel filtration and SDS-PAGE (Supplementary Fig. S7).

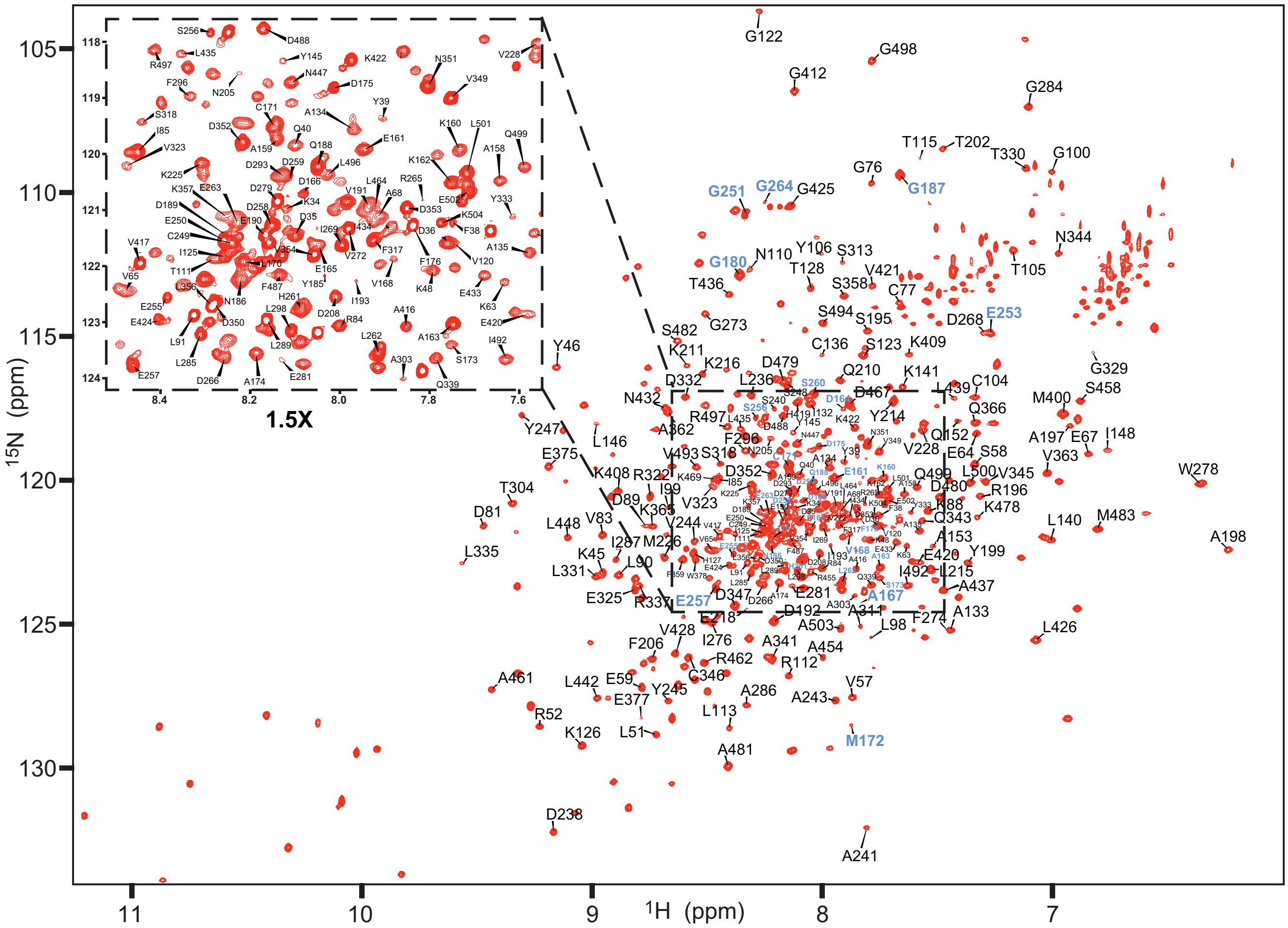

Figure S3. ^{1}H-^{15}N TROSY spectrum of FTO. The assigned backbone amide correlations of human FTO are labeled on the 800 MHz ^{1}H-^{15}N TROSY spectrum acquired at 30 °C. As expected, signals originating from highly disordered loops (residues 160-188 and 251-267) show poor chemical shift dispersion (blue labels). Of note, only 248 out of 426 observed NH correlations were assigned. The main cause for the low degree of unambiguous assignments is poor magnetization transfer from the backbone amide groups to the side-chain carbon atoms that resulted in low quality HNCB and HN(CO)CACB spectra.

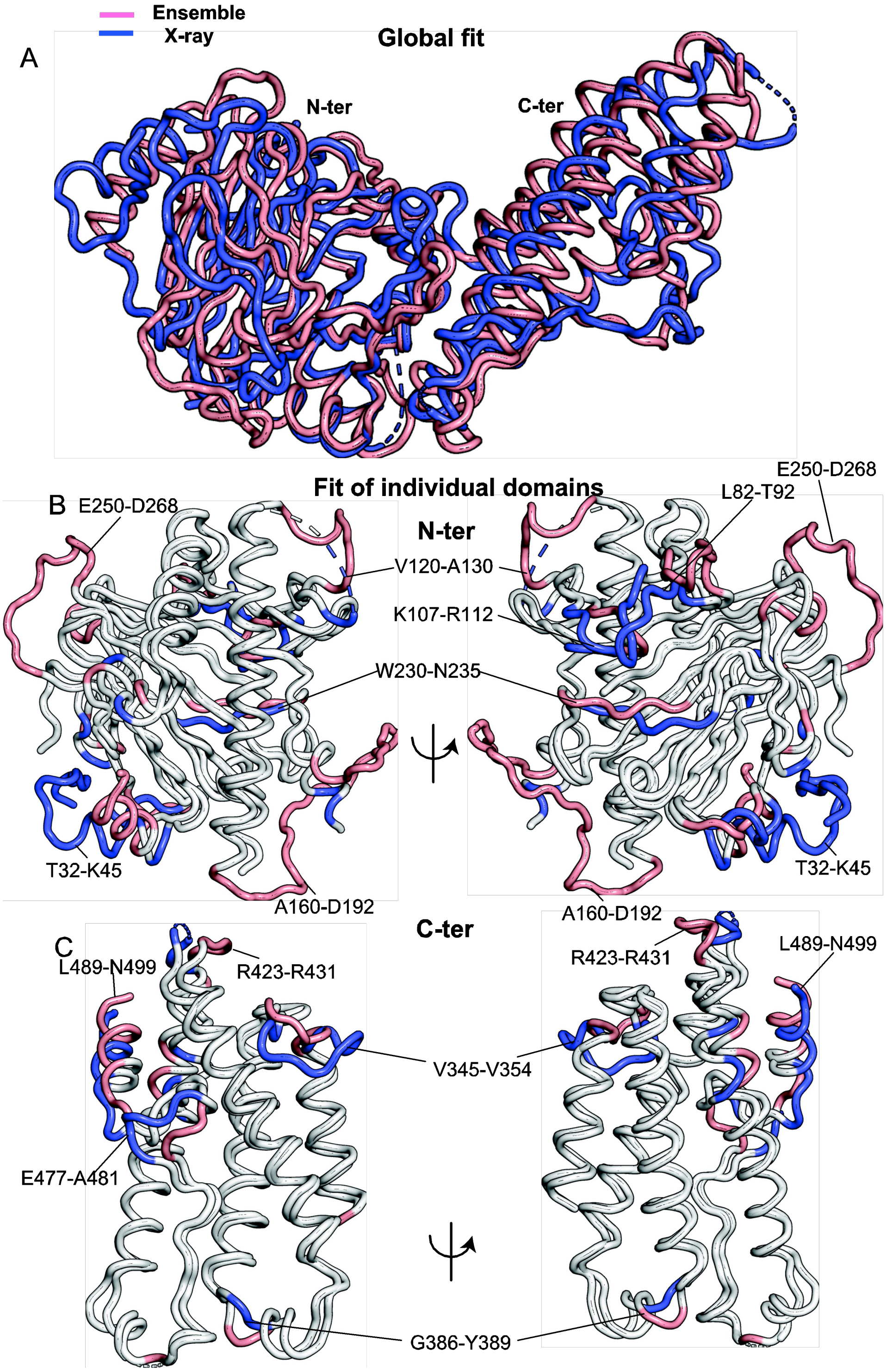

Figure S4. Structural differences between X-ray and representative structure. (**A**) The representative structure calculated over the FTO conformational ensemble (salmon cartoons) is overlaid on the X-ray structure of holo FTO (blue cartoons) (PDB code: 3LFM). In order to highlight local conformational changes, superimposition was also performed using the coordinate of the N- (**B**) and C-terminal domain (**C**) only. In (B) and (C), residues that can be superimposed with Cα displacement ≤ 3 Å (see Fig. 2G) are colored white.

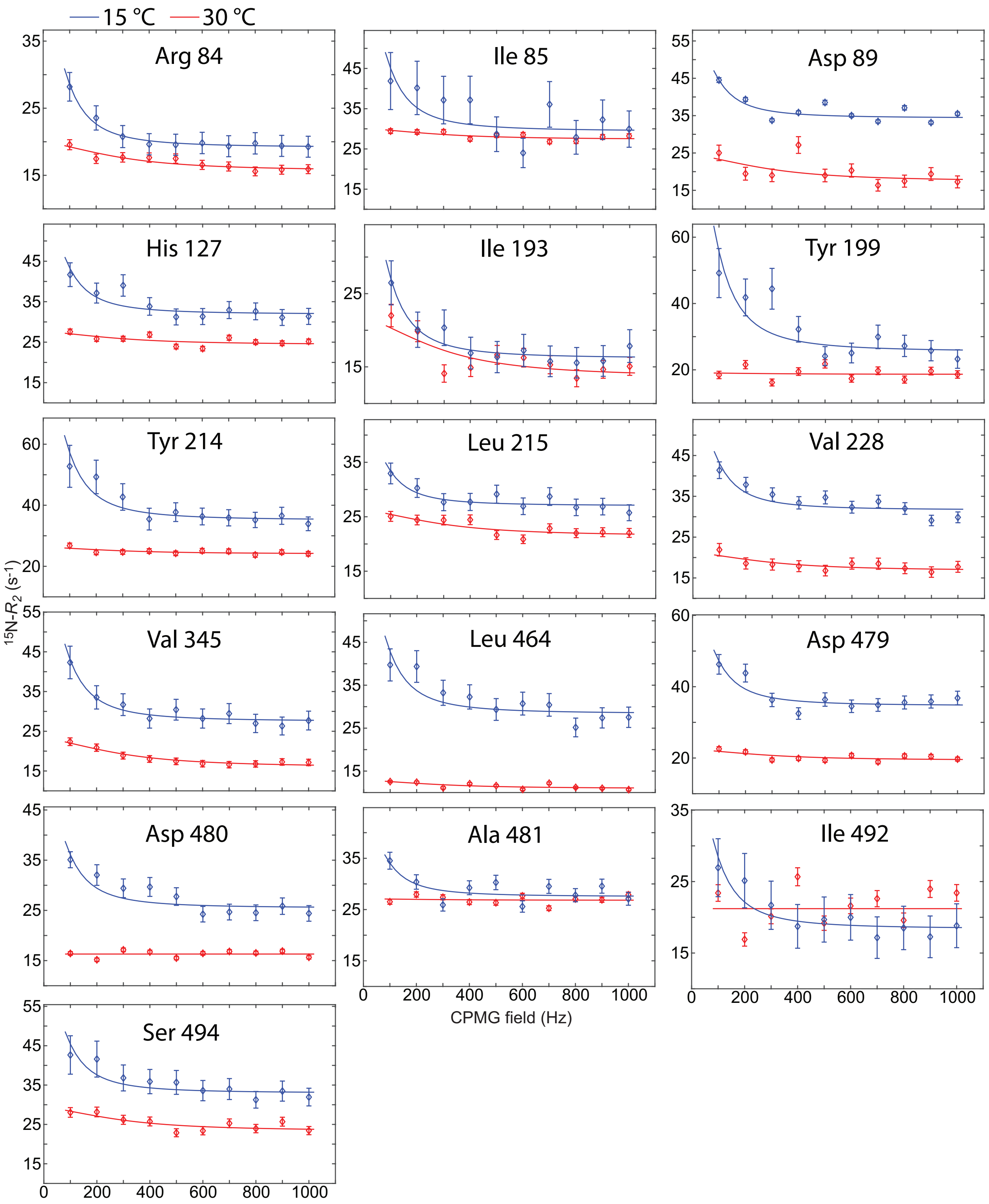

Figure S5. RD curves measured for apo FTO. The 800 MHz RD curves for apo FTO measured at 30 (red) and 15 °C (blue) were globally fit to a two-site exchange process using the Carver Richards equation. The optimized exchange rate constant (k_{ex}) is ~500 s^{-1} and ~100 s^{-1} at 30 and 15 °C, respectively. The experimental and modelled data are shown as circles and solid curves, respectively.

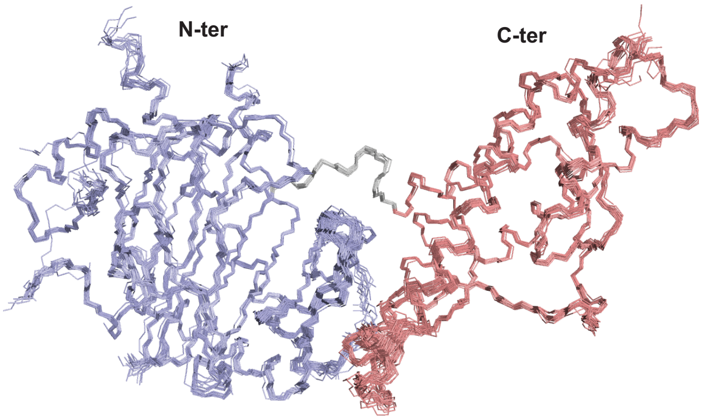

Figure S6. Conformational heterogeneity in the FTO crystal structures. Overlay of all the X-ray structures solved to date for human FTO (PDB codes: 3LFM, 4CXW, 4CXX, 4CXY, 4IDZ, 4IE0, 4IE4, 4IE5, 4IE6, 4IE7, 4QHO, 4QKN, 4ZS2, 4ZS3, 5DAB, 5F8P, 5ZMD, 6AEJ, 6AK4, 6AKW, 7CKK, and 7E8Z). The protein backbone is shown as lines. The N-terminal domain is colored light blue. The C-terminal domain is colored salmon. The linked is colored light gray.

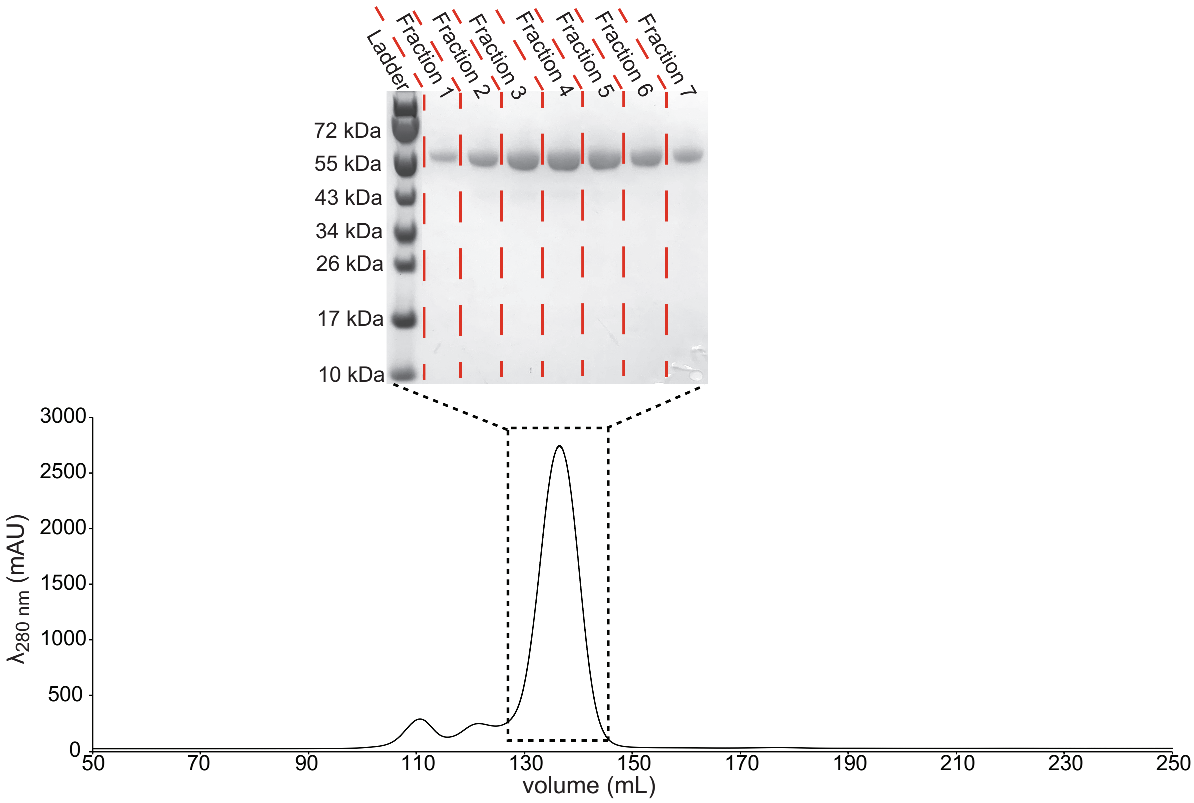

Figure S7. Purity of the selectively ^{15}N-labeled FTO samples. Superdex 75 elution profile and SDS-PAGE gel acquired after removal of the EIN fusion tag (see Experimental procedure) for a representative ^{15}N-labled FTO sample (the sample selectively labeled with ^{15}N Phe is shown). The protein elutes at the expected volume for enzymatically active FTO (dashed box) (1). Note that one lane between the Ladder and Fraction 1 was spliced off the gel.

References

1. Khatiwada, B., Purslow, J. A., Underbakke, E. S., and Venditti, V. (2020) N-terminal fusion of the N-terminal domain of bacterial enzyme I facilitates recombinant expression and purification of the human RNA demethylases FTO and Alkbh5. *Protein Expression and Purification* **167**, 105540
